# A strategy to combine pathway-targeted low toxicity drugs in ovarian cancer

**DOI:** 10.18632/oncotarget.5093

**Published:** 2015-09-22

**Authors:** Joe R. Delaney, Chandni Patel, Katelyn E. McCabe, Dan Lu, Mitzie-Ann Davis, Isabelle Tancioni, Tami von Schalscha, Alena Bartakova, Carley Haft, David D. Schlaepfer, Dwayne G. Stupack

**Affiliations:** ^1^ Department of Reproductive Medicine, UCSD Moores Cancer Center, La Jolla, CA, USA

**Keywords:** ovarian cancer, autophagy, combination therapy, adverse events, necramed

## Abstract

Serous Ovarian Cancers (SOC) are frequently resistant to programmed cell death. However, here we describe that these programmed death-resistant cells are nonetheless sensitive to agents that modulate autophagy. Cytotoxicity is not dependent upon apoptosis, necroptosis, or autophagy resolution. A screen of NCBI yielded more than one dozen FDA-approved agents displaying perturbed autophagy in ovarian cancer. The effects were maximized via combinatorial use of the agents that impinged upon distinct points of autophagy regulation. Autophagosome formation correlated with efficacy *in vitro* and the most cytotoxic two agents gave similar effects to a pentadrug combination that impinged upon five distinct modulators of autophagy. However, in a complex *in vivo* SOC system, the pentadrug combination outperformed the best two, leaving trace or no disease and with no evidence of systemic toxicity. Targeting the autophagy pathway in a multi-modal fashion might therefore offer a clinical option for treating recalcitrant SOC.

## INTRODUCTION

Targeted drugs with better safety profiles than traditional cytotoxic chemotherapeutics are increasingly available in the oncologist's armament. While use of single agent targeted therapies has shown only limited success [1–3], the lower toxicity of the drugs provides significant hope that logical combinations of these drugs may offer improved options relative to single drugs alone. Since each tumor might bear a fraction of cells resistant to any single therapy [4, 5], the use of monotherapies leaves resistant cells to initiate recurrent disease. Yet, problems of drug resistance in non-oncologic fields, including bacterial infection [6] and human immunodeficiency virus (HIV) [7], have been successfully approached with the use of multiple agents. Highly active antiretroviral therapy (HAART) was similarly developed to combat the high mutation rates present in HIV, and represents a remarkable success using three drug combinations.

In oncology, where the existing standards of care are highly toxic, there is little room for the addition of even those agents with modest toxicity. Targeting a single node on a signaling pathway with multiple drugs can be of benefit, though if drugs which target a single node are too similar in mechanism or chemical structure, resistance to one agent can confer resistance to others [8, 9]. Multinodal targeting may circumvent resistance, although side effects from such therapy are less predictable. However, *the risk of not finding efficacious regimens to treat cancer is similarly high*; every day this year, an average of 38 women will die of ovarian cancer from failed therapy, since there are no good options to treat the recalcitrant disease (American Cancer Society 2015 estimates).

SOC is among the most heterogeneous cancers [10], which may explain its abysmal five year survival rate of 17–39% (stage III/IV) (SEER 2004–2010 data). Early stage tumors are very hard to detect with high specificity [11], and SOC typically presents as disseminated disease. Standard of care chemotherapy consists of a platinum-taxane two drug regimen and results in an initial 2-year remission rate of 75% [12]. About half of these patients will recur. Novel agents targeting SOC rarely have sufficient impact to advance to Phase III trials.

Autophagy has been implicated as a mechanism in which dormant or otherwise chemotherapy resistant cells survive the initial platinum-taxane regimen [13–15]. However, autophagy agents have also been shown to induce death in SOC cell lines [16, 17]. Here, we examined both mode of death and cooperativity of the autophagy-modulating agents. While we noted an excellent cytotoxic effect of single agents that altered autophagy *in vitro*, the combinatorial use of relatively low toxicity agents revealed dramatic increases in cytotoxic efficacy in mouse tumor models. Based on these results, we suggest that it may be possible to create a combination that is able to treat the inherent heterogeneity of the tumor while harboring a favorable safety profile.

## RESULTS

### Identification of drugs that modulate autophagy

Targeting autophagy has been described as a method to enhance killing of subpopulations of SOC cells resistant to chemotherapy [13]. Since “autophagic death” can occur in parallel to classical forms of programmed cell death [20], such as apoptosis and/or necroptosis, and apoptotic and necroptotic initiation proteins are frequently inactive in SOC [18], we sought to investigate the mode of cell killing by agents which impact autophagy, with the hope of possible rapid translation to logical combination therapies within a clinical milieu.

**Figure 1:**
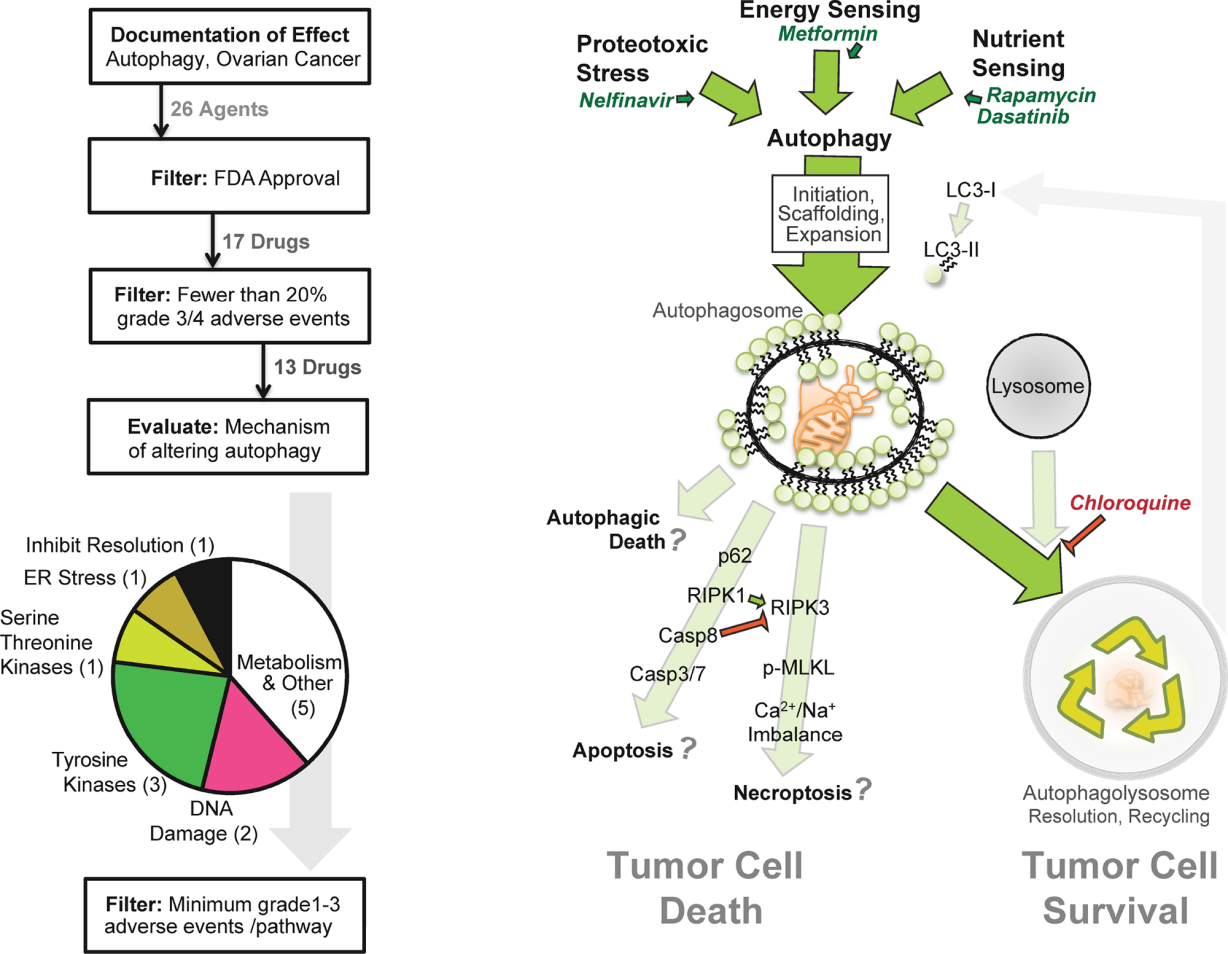
Strategy for pathway-based *in silico* drug selection (Left) Diagram outlining how many drugs were within each step of the drug selection process. The last filter selected a single drug from each mode of action based on documented minimal adverse events. (Right) Model of potential interactions between autophagy, autophagy drugs, and the cell death pathways necroptosis and apoptosis.

In a filtered search using the terms “autophagy” and “ovarian cancer” in PubMed and Google Scholar, 26 agents were found to impact autophagy in ovarian cancer (Table 1). Among these, 13 are FDA-approved and with a modest adverse event profile in patients (Figure 1). Surprisingly, however, these agents influence a repertoire of cellular processes including cellular metabolism, protein phosphorylation, proteotoxic stress, and lysosome acidification. This variation in target pathways suggests a broad interaction between SOC biological processes and autophagy. Within each molecular process, we then selected the drug with the lowest overall adverse events for further study. One was the DNA-targeted drug doxorubicin, which indirectly activates autophagy in response to genotoxic stress. The other five drugs were the ER stressor nelfinavir, the lysosomal acidification inhibitor chloroquine, the mTORC1 inhibitor rapamycin, the anti-diabetic metformin, and the Src family kinase inhibitor dasatinib (Figure 1).

**Table 1:** Drugs with evidence of affecting autophagy in ovarian cancer

						Most Frequent Adverse Events				
Drug	Target	Ref (PMID)	FDA approved?	Patent end date?	Autophagy stage	G1–2	G3	G4	Safety Reference	Notes
Nelfinavir	ER stress	19106637	Y	No patent	Initiation, compensation for ER stress	17%	-	-	FDA (NDA 21–503)	
Chloroquine	Lysosome	19033662	Y	No patent	Clearance	2%	-	-	PMID 10759574	
Dasatinib	Src family	20629079	Y	6/2020	Initiation	17%	5%		FDA (NDA 21–986)	
Rapamycin	mTORC1	19033662	Y	No patent	Initiation and expansion	35%	15%	2%	PMID 21752435*	Temsirolimus
Metformin	AMPK, LKB1	21532889	Y	No patent	Stem cells or AMPK/LKB1/initiation	8–13%	-	-	PMID 17638715	
Doxorubicin	DNA	22860102	Y	Equivalents	Initiation	37%	11%		FDA (NDA 50–718)	
Titanocene Y	DNA	23019413	N	-	Initiation	-	-	-		
Carboplatin	DNA	21743489*	Y	No patent	Initiation, protective autophagy	98%			FDA (NDA 20–452)	*cisplatin
5-fluorouracil	DNA, ER stress	22684338	Y	Equivalents	Initiation	33%	52%	42%	FDA (NDA 20–985)	
Diindolylmethane	ER Stress	22564965	N*	-	Initiation	0%	0%	0%	FDA (Docket 95s0316/sup0002)	*Supplement
3-methyladenine	BECN1	24817946	N	-	Initiation block, enhances cisplatin death	-	-	-		
Saquinavir	ER Stress	19147209	Y	11/2015	Initiation	11%	-	-	FDA (NDA 21–785)	
Fulvestrant	Estrogen receptor	22896656	Y	1/2021	Initiation	68%	10%		FDA (NDA 21–344)	
L-asparaginase	Glycosylation	22333033	Y	No patent	Initiation	28%	5%		FDA (125359 Orig1s000)	
Suberoylanilide hydroxamic acid	HDAC	21491416	Y	2/2025	Initiation	58%	4%		FDA (NDA 21–991)	
Bafilomycin	Lysosome	22564965	N	-	Clearance	-	-	-		
SB202190	MAPK	21853067	N	-	Clearance	-	-	-		
Decitabine	Methylcytosine	21491416	Y	Equivalents	Initiation	-	27%	93%	16532500 and FDA (NDA 205582)	
Paclitaxel	Microtubules	22430212	Y	Equivalents	Initiation	-	46%		FDA (NDA 20–262/S-024)	
Wortmannin	PI3K	21853067	N	-	Initiation		(toxic)		PMID 2527336	
Bortezomib	Proteasome	19584239	Y	10/2014	Proteasome disruption	100%	61%	14%	FDA (NDA 21–602)	
MG132	Proteasome	23270461	N	-	Proteasome disruption	-	-	-		
H2O2	ROS	23047606	N	-	Initiation	-	-	-		
FTY720	S1P receptor	20935520	Y	2/2019	Initiation, siBECN1 and siLC3 block effect	9%	-	-	FDA (NDA 22–527)	
Arsenic trioxide	Unknown	22919067	Y	11/2018	Initiation (less pAkt)	75%	13%		FDA (NDA 21–248)	
Withafarin A	Vimentin	22860102	N	-	Initiation	-	-	-		

### Autophagy-modulating agents do not induce classical cell death pathways

“Autophagic death” occurs via several mechanisms, yet it has been suggested to be a misnomer, since autophagy often accompanies other forms of cell death [21]. It therefore becomes important to distinguish between causation, such as when autophagosomes act as an intracellular scaffold for caspase-8 activation [22], vs. simple co-occurrence of programmed cell death and autophagy. In many cases, the autophagic response is elicited to try to correct the stress inducing programmed cell death. One indicator of cellular stress is sequestosome, or p62, an adaptor that targets ubiquinated proteins to autophagosomes, though it may also recruit caspase-8 [23] or RIPK1 [24] as part of apoptotic or necroptotic pathways respectively (Figure 1).

**Figure 2:**
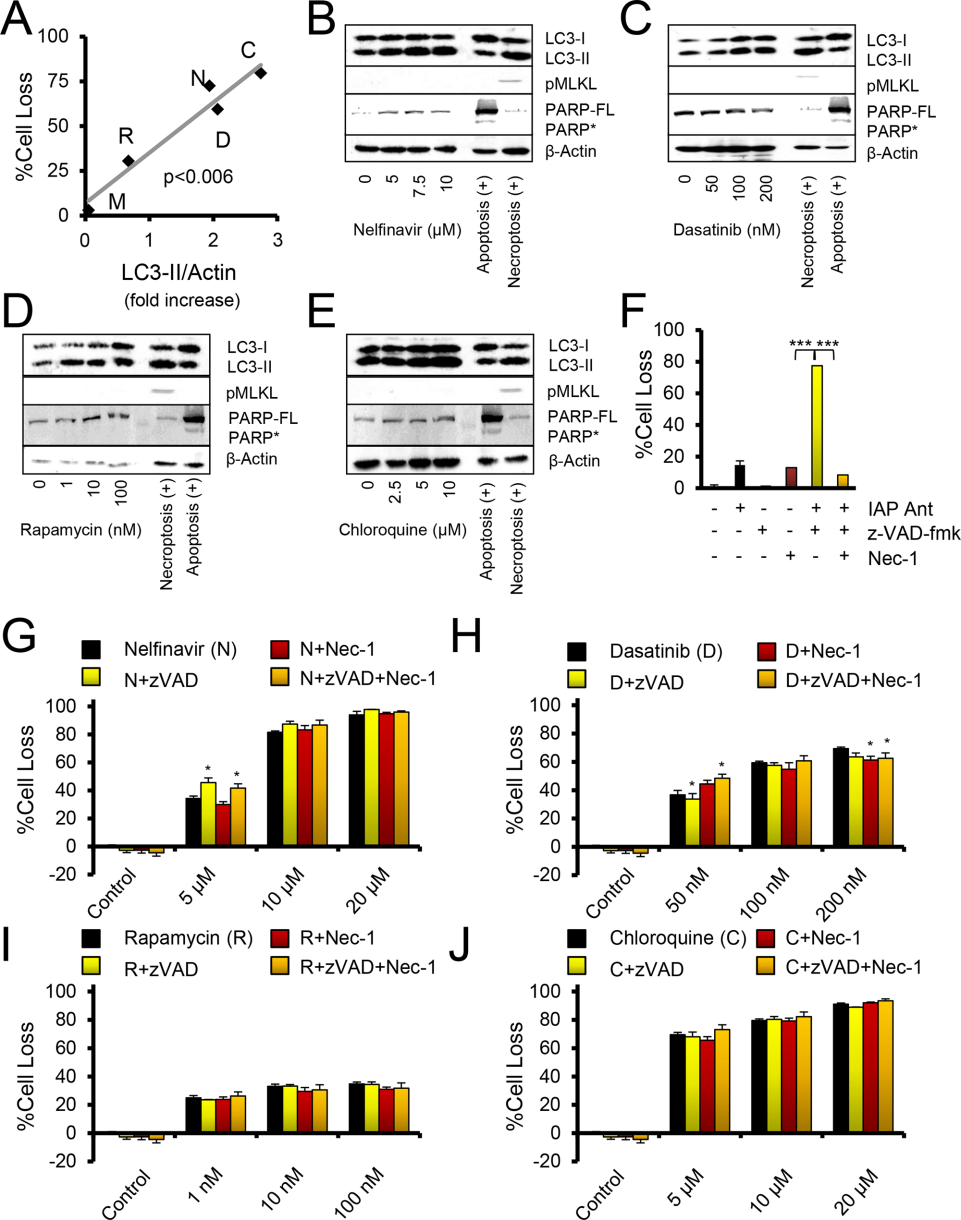
Autophagy drugs act independently of apoptosis and necroptosis **A.** Comparison of LC3-II immunoblot levels to %Cell Loss. Doses were: chloroquine (C, 10 μM), nelfinavir (N, 10 μM), rapamycin (R, 10 nM), dasatinib (D, 100 nM), metformin (M, 10 μM). *P* value is a Pearson's correlation test. **B–E.** Immunoblots of OVCAR3 cells treated for 24 hours with the indicated drugs. The necroptosis positive control is OVCAR3 cells treated with Birinapant and z-VADfmk (20 μM). The apoptosis positive control is 293T cells treated with cisplatin at 10 μM. PARP* indicates cleaved PARP, PARP-FL indicates full length PARP. pMLKL is pS358-MLKL. **F–J.** Crystal violet proliferation assay of OVCAR3 cells grown for 48 hours in the presence of the indicated drugs. %Cell Loss indicates difference of treated cells to that of untreated, maximally proliferating controls. Nec-1, necrostatin, was used at 30 μM. *p < 0.05, by *t*-test to autophagy drug of same dose. (F) Control crystal violet proliferation assay done within the same assays as other cell loss panels, where IAP Ant is the SMAC mimetic Birinapant at 100 nM, which produces necroptosis when caspases are inhibited by z-VADfmk, and the necroptosis can be suppressed by addition of Nec-1. ***p < 0.001 by *t*-test. All error bars are s.e.m.

To evaluate cell death in the context of autophagy inducing drugs, we drugged OVCAR3 cells within the range found in monotherapy treated patients’ blood (Table 2). Although many ovarian cancer cell lines are not genetically representative of SOC [25], the OVCAR3 line closely aligns with TCGA-documented SOC, has a deep accompanying dataset (>1000 publications), and behavior similar to cisplatin resistant cells derived from recurrent patients [26]. In agreement with prior literature, we noted an induction of autophagosomes upon drug addition. This was indicated by an increase in steady state LC3-II compared to β-actin, not the LC3-II to LC3-I ratio, which may have confounding signals from protein turnover during autophagy as well as LC3-I induction independent of autophagic flux [27]. Interestingly, a correlation of LC3-II/β-actin with cell loss (*p* < 0.006, Figure 2A) was observed among four of the five drugs, while metformin was observed to be relatively ineffective (<5% cell loss) in OVCAR3 cells.

**Table 2:** Patient blood levels of autophagic drugs

	MW	Peak		Molarity	Trough		Molarity	Reference (PMID)
Metformin	165.62	1600	ng/mL	9.66E-06	273	ng/mL	1.65E-06	22864111
Chloroquine	515.86	4500	ng/mL	8.76E-06	600	ng/mL	1.16E-06	3289601
Nelfinavir	663.89	3820	ng/mL	9.94E-06	125	ng/mL	3.27E-07	NDA 21-503
Rapamycin	914.2	30	ng/mL	1.15E-07	23	ng/mL	8.80E-08	9721433
Dasatinib	488.1	129	ng/mL	2.64E-07	8.7	ng/mL	1.78E-08	22837181

Strikingly, none of the drugs induced PARP cleavage (as observed in apoptosis, and sometimes in necroptosis) or phosphorylation of MLKL (a marker of necroptosis) (Figure 2B–2E). The results suggested that cell loss was not associated with apoptotic or necroptotic cell death. To investigate this further, we employed pharmacologic agents to directly modulate the apoptotic and necroptotic pathways. The inhibitor z-VAD_fmk_ blocks cysteine protease activity, compromising caspase activation and attenuating apoptosis, and can potentiate necroptosis. Necrostatin is an inhibitor of RIPK1 activity and blocks many forms of necroptosis. Necrostatin and z-VAD_fmk_ both proved able to modulate death induced by a small molecule antagonist of the IAPs (Figure 2F). In this case, z-VAD_fmk_ potentiated necroptotic death, while necrostatin rescued cell survival [26]. However, neither necrostatin nor z-VAD_fmk_ showed more than 10% rescue in cell loss induced by nelfinavir, chloroquine, dasatinib, or rapamycin (Figure 2G–2J). In summary, all agents which created autophagic stress, as represented by dasatinib, rapamycin, and nelfinavir, promoted cell death. However, inhibition of autophagy by chloroquine also promoted death. Thus, death did not appear to occur as a result of autophagy per se, although LC3-II expression increased along with cytotoxicity.

### Combinatorial autophagic stresses enhance SOC cell death

**Figure 3:**
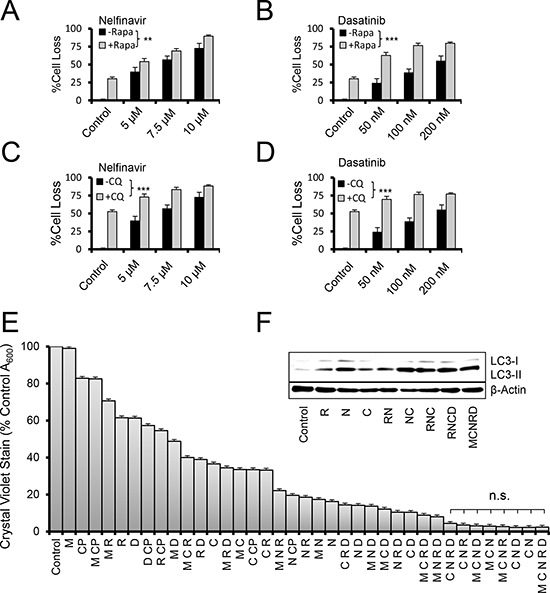
Autophagy aggravation and inhibition increase cell loss in combination therapy **A–D.** Crystal violet proliferation assay of OVCAR3 cells grown for 48 hours in the presence of the indicated drugs. %Cell loss indicates difference of treated cells to that on untreated, maximally proliferating controls. +/– Rapa indicates the presence of rapamycin dosed at 100 nM. +/– CQ indicates chloroquine added at 5 μM. **p < 0.01, ***p < 0.001 by one-sample *t*-test comparing the difference of the drug pair and the most active single drug to the null difference of zero. **E.** Two day crystal violet proliferation assay depicting a fully factorial combination screen for the five autophagy drugs metformin (M, 10 μM), rapamycin (R, 10 nM), dasatinib (D, 50 nM), chloroquine (C, 9 μM), and nelfinavir (N, 9 μM). Doses represent peak (M,C,N) or trough (R,D) drug concentrations found in patient blood levels. Cisplatin (CP, 5 μM) was also used for comparison. n.s. indicates sample comparisons are *p* > 0.05 by student's *t*-test. All error bars are s.e.m. **F.** Immunoblot of OVCAR3 cells treated with the indicated drugs for 24 hours. Doses were R 100 nM, N 5 μM, C 2.5 μM, D 100 nM, and M 10 μM, chosen for their dynamic range.

We then tested whether combinations of different agents resulted in any alteration of the cell death observed. Interestingly, the addition of rapamycin to other autophagy-initiating agents produced significantly additive effect on cell death (Figure 3A, 3B). Similar results were obtained when chloroquine was combined with these agents (Figure 3C, 3D), raising the notion that autophagy occurred in response to an autophagic stress on the cells.

Higher order combinations of the five drugs produced, in general, increasing efficacy (Figure 3E). However, the combination of chloroquine and nelfinavir (CN) was also among the most potent at inducing cell death, and the only two-drug combination found to do so in a 48 hour time-frame. Nonetheless, immunoblot analysis of LC3-II levels at 24 hours proved a reasonable surrogate of cell death at 48 hours (Figure 3F); peaking in those combinations that had maximal cytotoxic activity. Similar efficacy was found in other SOC models ID8ip, OVCAR8, and PS#3971, and did not depend on cisplatin sensitivity (Supplementary Figure S1).

### Combinatorial autophagic stresses enhance LC3 and p62 punctae

**Figure 4:**
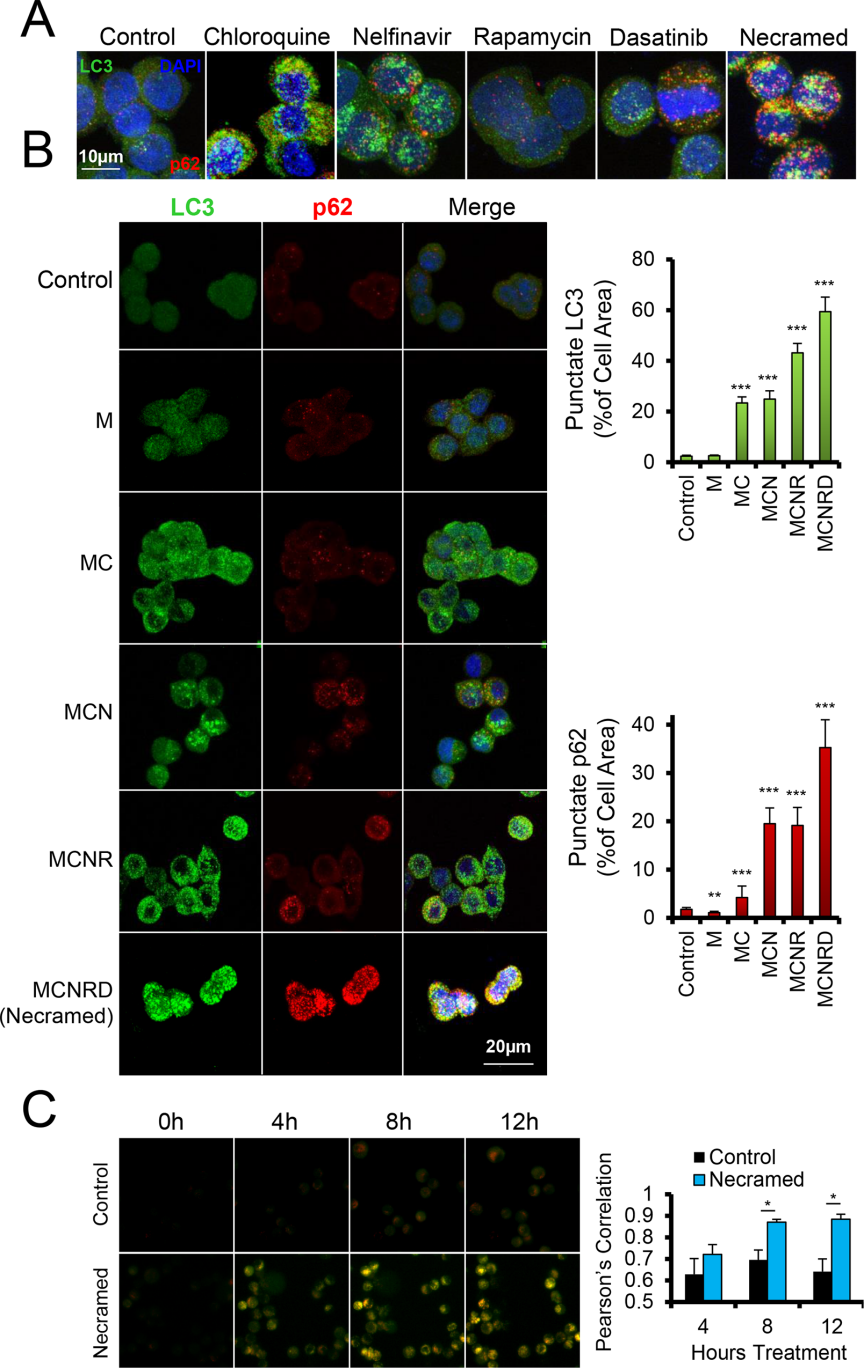
Measurement of dysregulated autophagy by fluorescent microscopy OVCAR3 cells were treated with the indicated drugs for 24 hours and then fixed and immunostained for p62 (red) and LC3 (green), with a DAPI costain (blue). Confocal z-stacks were flattened for image analysis to capture all punctate area. **A.** Doses were: chloroquine (10 μM), nelfinavir (10 μM), rapamycin (10 nM), dasatinib (50 nM), and all were combined for Necramed. **B.** Similar immunostaining as in (A) Punctae were quantified for size and number per cell by ImageJ with at least 40 cells per condition. LC3 channel was analyzed for autophagosomes, and the p62 channel for sequestosomes. Doses were same as in (A), but with chloroquine and nelfinavir reduced to 5 μM. *p < 0.05, ***p < 0.001 by *t*-test. All error bars are s.e.m. **C.** OVCAR3 cells with a virally integrated mCherry-GFP-LC3 construct were studied by live microscopy, for the number of hours indicated following Necramed (doses as in (A)) or control treatment.

To evaluate whether LC3-II accumulation corresponded to the presence of autophagosomes, we next stained cells to evaluate the relocalization of LC3 from diffuse cytosolic distribution to punctae indicative of autophagic vesicles. Following treatment with the panel of agents, alone (Figure 4A) or in combination (Figure 4B), we found that the relative measure of LC3 positive punctae was in agreement with the immunoblot data.

In parallel, we evaluated the presence of p62/sequestosome as a reporter of the cell's sensation of proteotoxic stress. Autophagy normally clears p62 when functioning properly, however stress that induces autophagy also induces p62 expression [14, 28]. Punctate p62 can therefore indicate an over-stressed autophagy system [29]. Nelfinavir or chloroquine treatment elevated punctate p62, while rapamycin and dasatinib had a more modest effect, and metformin slightly reduced p62 but did not alter punctate LC3 compared to controls. However, when drugs were used together, p62 accumulated, suggesting the presence of an ongoing, unrelieved proteotoxic stress despite the overt induction of autophagy. The p62 and LC3 punctae reached a maximum signal upon treatment with all five autophagy drugs, which we term for simplicity Necramed (Nelfinavir, chloroquine, rapamycin, metformin, and dasatinib).

To further test the quality of autophagy, we used a dual labeled mCherry-GFP-LC3 reporter system [30]. Autophagolysosome formation quenches the GFP signal due to acidification, which mCherry is resistant to and remains fluorescing red. Yellow punctae thus primarily represent autophagosome structures which have not successfully fused with functional lysosomes and therefore have not cleared enclosed damaged organelles and proteins. As expected with a drug combination containing chloroquine, LC3 punctae using this reporter were primarily yellow, stalled autophagosomes, which accumulated during Necramed treatment (Figure 4C).

### Evaluation of combinations of cell stressors *in vivo*

**Figure 5:**
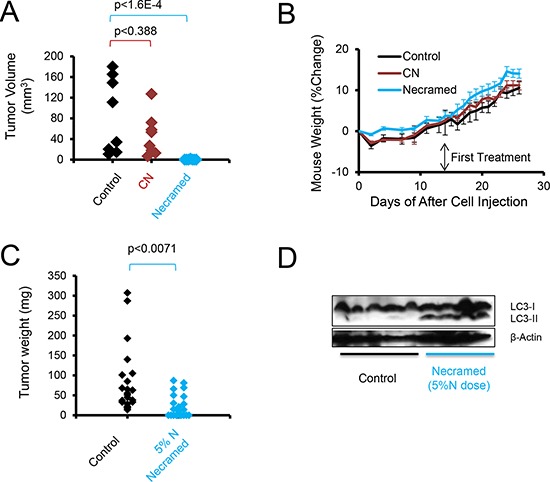
Autophagy combination therapy suppresses SOC *in vivo* **A.** C57BL/6 immunocompetent mice were injected IP with 3 × 10^6^ ID8ip-mCherry cells. After two weeks, mice were orally gavaged daily with control, Necramed, or with chloroquine (C) and nelfinavir (N) only. Following two weeks of treatment, mice were euthanized and mCherry fluorescent tumors quantified. **B.** The weight profiles of mice in (A) Error bars are s.e.m. **C.** A patient derived xenograft model, using ascites cells from a recurrent platinum-insensitive patient, was established by injecting 3 million PS#3971 cells IP into nude mice. One week following injection, mice were gavaged daily with control or 5%N Necramed (Nelfinavir reduced to 25 mg/kg), for 32 days. Mice were euthanized and macroscopic tumors dissected and weighed. Wilcoxon rank sum test was used to statistically compare groups. **D.** Immunoblots of pooled tumors dissected from each mouse in (C).

Given that the cytotoxicity *in vitro* was similar between all five autophagy drugs and the simple combination of chloroquine and nelfinavir (Figure 3E), despite apparent differences in proteotoxic stress induced in the cells, we wondered whether these two approaches might act similarly *in vivo*. To test this, the syngeneic, ID8ip-mCherry [19] cell model was used. The literature on the doses of these autophagic drugs to use *in vivo* varies, so we opted to use a standard, FDA recommended method of mouse-human dose adjustment calculations [31] which focuses on surface area, blood physiology, and metabolism (Table 3). In this case, peritoneal tumors were allowed to develop for two weeks, and then mice were treated daily with control solution, chloroquine and nelfinavir (CN), or with the combination of all five agents (Necramed) for 14 days. Interestingly, and despite the fact that these regimens yielded similar efficacy *in vitro* (Figure 3E), there was a marked difference *in vivo* (Figure 5A). Tumors could be macroscopically detected in the CN group, and did not significantly differ than those from untreated control mice. In dramatic contrast, there were no macroscopic tumors detectable in the Necramed group, although 1/8 mice had a residual island of cells microscopically detectable on an ovary (1.7 mm^3^, Figure 5A). There were no overt indications of toxicity either by gross visual examination or by weight loss in either the CN or Necramed groups (Figure 5B). Consistent with the initial screening strategy to select drugs with minimal adverse effects (Figure 1) there were no gross histological changes in Necramed-treated mice (Supplementary Figure S2). Finally, no signs of toxicity in blood panels were observed in Necramed-treated mice relative to untreated mice (Supplementary Table S1). Thus, despite a potent anti-tumor activity, no overt systemic toxicity was associated with Necramed treatment.

**Table 3:** Human to mouse dose conversions

Drug	Human mg/kg*	Human Reference	Mouse mg/kg**	Necramed mg/kg (mouse)	Mouse reference	Reference mouse dose (mg/kg)
Metformin	16.7	Metformin OvCa clinical trial	205.6	150	PMID 22864111	150
Chloroquine	8.3	PMID 19326448	102.8	30	PMID 19033662	50
Nelfinavir	41.7	Drugs@FDA	513.9	500 (100%)	PMID 22664238	5
Rapamycin	0.3	PMID 22872575	3.3	2.24	PMID 23014526	10
Dasatinib	1.7	Sprycel dockets	20.6	4	PMID 20629079	10

* All human mg/kg are from oncology clinical trials, except for Nelfinavir

** Dose conversions from human mg/kg, using calculation from reference PMID 17942826

### LC3-II is detected in tumors treated with the necramed combination

A problem with the observed efficacy was that it did not permit the evaluation of LC3-II increases with treatment, as there were no substantial tumors left amenable to immunoblot analysis. To remedy this and test another tumor model, we next treated mice with Necramed in which we titrated nelfinavir to 5% (25 mg/kg, 1/10^th^ the ID8ip model dose) of a dose converted from human use into a mouse dose [31], following seeding with the human PS#3971 tumor model. The reduced-nelfinavir combination impacted the distribution of tumors *in vivo* (Figure 5C) but enough tumor remained to allow for detection of increased LC3-II/β-actin in treated mice (Figure 5D). Altogether, the results supported the notion that LC3-II acted as a reporter for Necramed activity.

## DISCUSSION

We find that a patient-data centered screen of previously studied drugs with documented evidence of affecting a defined pathway in ovarian cancer permits one to derive a safe and efficacious combination. Here, agents were not selected based on a history of oncologic use, but rather on reported alteration of autophagy. Our data supports a limited impact of single agents, since even the best two agents combined lacked significant effect in our aggressive mouse model. In contrast, reducing one effective component from 250 mg/kg to 25 mg/kg retained significant activity when used as part of the five drug combination therapy.

The agents that came through the screen are varied in targets and function. Dasatinib, originally designed as an inhibitor of Bcr-Abl, also targets tyrosine kinases such as Src which act upstream of Akt and mTOR [16] (Supplementary Figure S3). Rapamycin, which failed as a cancer monotherapy, acts proximal to the Becn1/Ulk1/Vps34 autophagy initiation complex by inhibiting mTORC1 [32]. The other agents are rarely or never indicated for cancer therapy. Nelfinavir is an inhibitor of HIV-1 protease [33], but induces ER stress [34, 35], reduces proteasome function [36], and may weakly inhibit an array of kinases including Akt [37]. Metformin is used in diabetes mellitus; it is thought to trigger AMPK upstream of the Becn1/Ulk1/Vps34 complex [38], though it has additional metabolic effects [39] that may impact stem cells [40] and contribute to its documented capacity to improve survival of ovarian cancer patients [41]. Chloroquine is unique in its capacity to block autophagy. More specifically, it blocks late events, preventing fusion of autophagosomes with lysosomes and autophagic ‘flux’ [42, 43]. Developed as an antimalarial, it has been used in a small number of cancer clinical trials [44], and derivatives of chloroquine are currently of interest in a number of cancers.

Although drug combinations are routinely prescribed in antimicrobial applications, oncologists avoid combinations in cancer chemotherapy, since they frequently involve pushing the limits of toxicity in the attempt to eradicate cancer and may not offer further benefit [45] if their mechanism of action is similar. Therefore, to best allow for combining these agents with minimal chance of detrimental side effects, we focused this screen on those drugs with the lowest number and severity of adverse event reports. Consistent with this, we did not observe systemic toxicity, even in mice treated with all five agents. In retrospect, this is perhaps not surprising; a ‘typical’ cancer patient is associated with an aging demographic that frequently takes medications for one, or many, unrelated non-oncologic indications.

**Figure 6:**
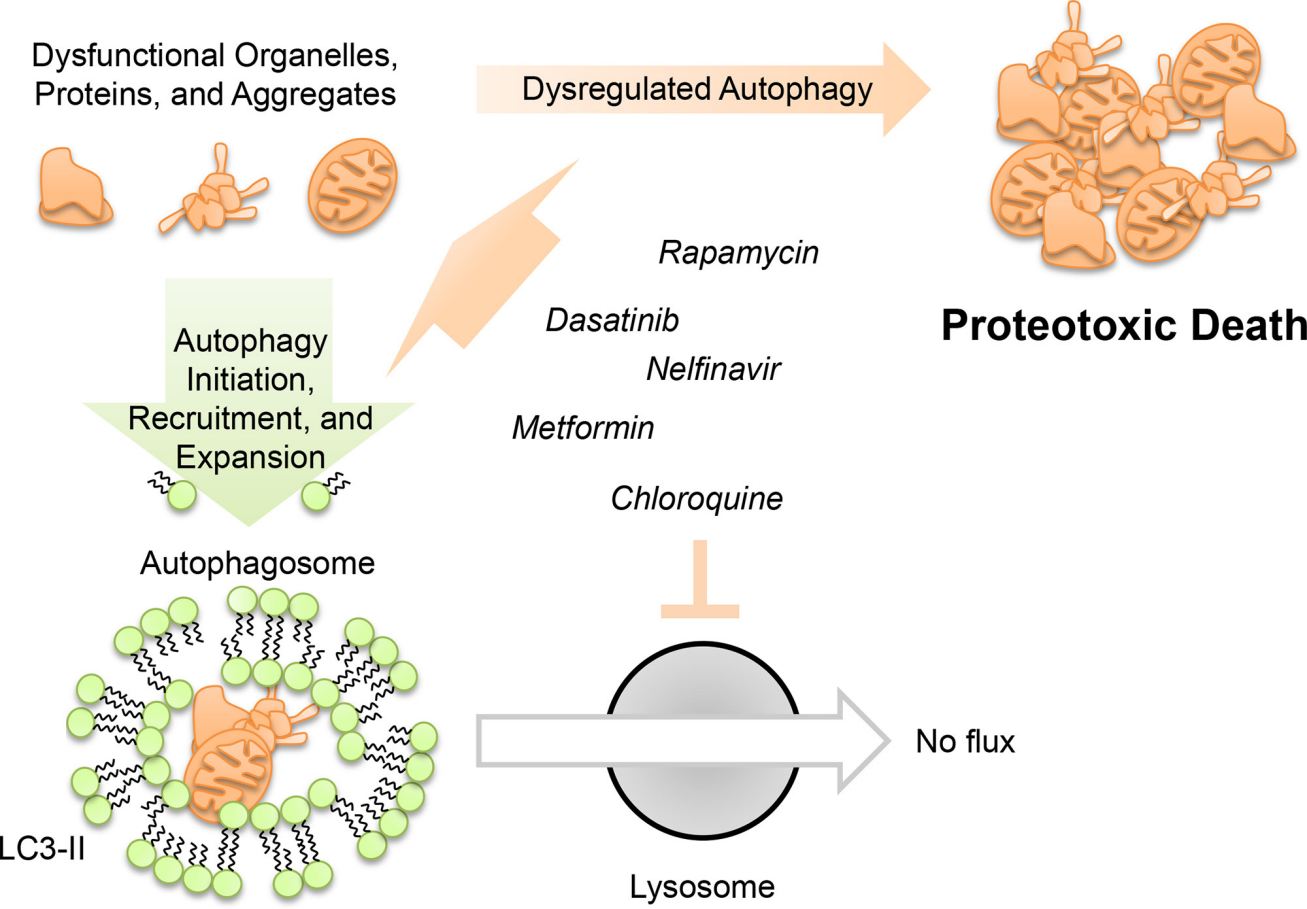
Model for dysregulated autophagy mediated proteotoxicity in ovarian cancer

This screen targeted drugs that modulate autophagy, which is considered both a cancer cell survival factor [46, 47] and drug resistance [15] factor in ovarian cancer. Although we induce autophagosome formation, it does not appear to be the instrument of cell death. In fact, we make the distinction that arresting autophagy did not ameliorate death, but rather enhanced it (Figure 3). Together with prior data, this supports the concept that autophagy may represent a mechanism by which tumor cells resolve inherent or induced stresses [48, 49]. Thus, combining agents which initiated cell stresses that activate autophagy with one that blocked resolution of autophagy acted cooperatively to kill cells (Figure 6). In our study, we found no evidence of apoptosis or necroptosis. Considering that resistance to apoptosis is a common hallmark of cancer [50] it may be that alternative mechanisms of cytotoxicity, such as proteotoxicity, are worth considering in a therapeutic context.

Can these results translate into the clinic? The focus on limited adverse events based on existing patient data, some from very large cohorts [51], appears promising for the perspective of a Phase I trial. Hesitation to use drug combinations is waning. Doxorubicin is a standard second/third line treatment for SOC, usually given in a less toxic pegylated liposomal form as Doxil^®^, and has been used in combination with anti-HIV HAART therapy for Kaposi's sarcoma. HAART typically consists of a minimum of a three drug combination and has included Nelfinavir. Doxil^®^, with its standard use and relatively favorable side effect profile, may be an ideal candidate for the chemotherapy which uses Necramed in an adjuvant setting.

How might Necramed itself be administered? We delivered all drugs by gavage, since in human patients all drugs are delivered orally. Theoretically, a slight delay in the administration of chloroquine might optimize the effects of the other collaborative, stress-inducing agents. However, we co-administered all drugs, at least initially, to simplify patient compliance and adherence. Since concomitant dosing of all drugs was successful and tolerable in our mouse models, simultaneous dosing is a viable clinical option. Drug dosage can be immediately reduced within individual patients if side effects are limiting, as may be determined in a Phase I clinical trial. Given the daily loss of more than three dozen women in the US to ovarian cancer, the risk of avoiding efficacious combination therapies is very high, and can outweigh the potential risks of combinatorial side effects.

## MATERIALS AND METHODS

### Reagents

All cells were grown in RPMI (Life Technologies) supplemented with 2 g/L glucose, nonessential amino acids, sodium pyruvate, and 10% FBS (Omega Scientific). Cells were always incubated at 37°C in a water jacketed incubator with 5% CO2. *Antibodies*. For western blots, Actin (Sigma #A5441-.2ML), LC3 (Novus Biologicals #NB100-2220), PARP (BD Biosciences #51-6639GR), pS358-MLKL (abcam #ab187091) were used. Secondary HRP-conjugated antibodies were anti-rabbit (Jackson ImmunoResearch #211-032-171) anti-rat (Life Technologies #619520), or anti-mouse (Jackson ImmunoResearch #115-035-003). *Drugs*. Cisplatin (Teva Pharmaceuticals, US, 1 mg/ml injectable) was obtained by the Moores Cancer Center pharmacy. Metformin (VWR, cat# 89147-892), chloroquine phosphate (Fisher Scientific, ICN19391910), rapamycin (LC Labs, cat# R-5000), dasatinib (LC Labs, cat# D-3307), and nelfinavir mesylate (Creative Dynamics Inc, special order except in syngeneic mouse model, which used pulverized Viracept tablets obtained from the Moores Cancer Center pharmacy) were purchased in powdered form. PEG400 for *in vivo* drug vehicle was from Spectrum Laboratory Products (#TCI-N0443-500G) and mixed with sterile saline (Teknova, #S5812). Patient consent was obtained for scientific use and publication of the OV#3971 patient derived ovarian cancer [18].

### Crystal violet proliferation assays

Cells (2,500–5,000/well) were seeded onto 96 well TC treated plates, allowed to attach, and then treated with drugs or control vehicle in a total volume of 100 μl. Plates were placed at 37°C for 48 hours (unless otherwise indicated). Media was removed and cells were washed once with 125 μl PBS. PBS was then removed and 50 μl crystal violet stain (0.11% crystal violet, 0.17M NaCl, 22% MeOH, in water) was added. After 30 minutes room temperature staining, stain was removed and 125 μl PBS was added as a wash. Supernatant was carefully removed to minimize cell disturbance but maximize removal of unbound crystal violet. Plates were then dried at 37°C for one hour without lid and 85 μl MeOH was added to solubilize the crystal violet. Absorbance was read at 600 nm to determine cell density. Percent growth inhibition (%Cell Loss) was calculated using the formula: 100-(100*AbsDrug/AbsControl). This reflects loss from either death or impaired proliferation.

### Western immunoblotting

Cells (3 × 10^6^) were seeded on 10 cm plates, allowed to adhere for 16–24 hours, and treated with drugs or control for 24 hours at 37°C. 300–400 μl iced RIPA buffer (supplemented with a protease inhibitor cocktail (Sigma-Aldrich), 2 mM sodium orthovanadate, and 50 mM NaF) was added to lyse the cells (15 minutes, room temperature) at which point cells were collected using a cell lifter (Fisher Scientific). Lysates were spun at 10,000 g for 10 minutes at 4°C and supernatant saved and quantified by BCA assay (Pierce #23235). 30 μg protein was loaded per well of a 15% SDS-PAGE gel and transferred onto a PVDF membrane. Membrane was blocked in 5% dry milk (Genesee Scientific, #20-241). Primary antibodies were used at 1:1000 dilution, and secondary HRP-conjugated antibodies were used at 1:5,000 dilution. HRP substrate and enhancer was used at 600 μL per membrane (Super Signal West Dura Extended Duration Substrate, Pierce # 34075).

### Immunofluorescence

Coverslips coated with 2 μg/ml fibronectin were placed into non-TC treated 6 well plates. Excess fibronectin was removed with PBS washes. 10,000 cells were seeded directly onto the coverslip and allowed to adhere for 30 minutes at 37°C. Media with drugs or vehicle was then added to a total volume of 3 ml. Cells were incubated at 37°C for 24 hours. Media was removed and 1 ml 4% paraformaldehyde in PBS was added for 15 minutes at room temperature. Supernatant was aspirated and cells were washed once with PBS. PBS was aspirated and 2 ml 0.1% Triton in PBS was added for intracellular permeabilization. After 2 minutes, supernatant was aspirated and PBS wash performed. Cells were then blocked in 2% BSA for 30 minutes at room temperature and then a PBS wash performed. Primary antibodies were then added in 2% BSA (1:1000 dilution for LC3, 1:500 dilution for p62) and incubated for 90 minutes at room temperature. After three PBS washes, secondary antibodies were added in 2% BSA with DAPI (1.5 μg/ml) at a 1:1000 dilution for 90 minutes. Three more PBS washes were performed, and then one final ten minute PBS wash performed. PBS was aspirated and coverslips were mounted on glass slides using 30 μl Vectashield (Vector Laboratories #H-1400). Cells were imaged on a Nikon confocal microscope, creating z-stacks to permit punctae quantitation. Flattened z-stacks were used to quantify punctae in ImageJ by MaxEntropy thresholding.

### Autophagic flux microscopy

OVCAR3 cells with mCherry-GFP-LC3B (Addgene, plasmid # 22418) retroviral integrants were seeded (100 k cells) on a 12 well fibronectin treated (2 μg/ml) glass bottom plate and allowed to adhere overnight. Fields of view were set on an Olympus IX51 microscope outfitted with an environmental apparatus allowing for tissue culture (37°C and 5% CO_2_) conditions. Drugs were added and then cells imaged for mCherry and GFP fluorescence every 4 hours.

### Mouse cancer models

All animal protocols were approved by the UCSD IACUC; appropriate regulations were followed during experimentation on animals. *Syngeneic model*: 3 × 10^6^ mCherry labeled ID8ip cells [19], which have been passaged in the peritoneal cavity, were injected into syngeneic female C5BL/6 mice at 10 weeks of age. Eight mice of equal mean weights were used in each group. 14 days after injection, the control group received daily (7x/week) vehicle gavage injections (50% PEG400), the CN group received daily gavages of chloroquine (30 mg/kg) and nelfinavir (250 mg/kg, from pulverized Viracept tablets) in 50% PEG400, and the Necramed group received daily gavages of chloroquine (30 mg/kg), nelfinavir (250 mg/kg), rapamycin (2.24 mg/kg), metformin (150 mg/kg), and dasatinib (4 mg/kg) in 50% PEG400. Mice were monitored daily for distended abdomens following the first treatment injections. All mice were euthanized when ascites formation produced visible discomfort to control animals, which occurred after 14 days of treatment (28 days since cell injection). The peritoneum of the mice was exposed and any visible nodules on the peritoneum wall were surgically dissected along with the liver and ovaries. These tissues were then imaged with the OV100 Small Animal Imaging System (Olympus). Brightfield, GFP, and mCherry channel information were collected and only red fluorescent (but not green autofluorescent) punctae area was quantified in ImageJ. Fluorescent area was mathematically converted into tumor volume assuming spherical shape of the tumor and circular shape of the fluorescent area with the equations: area = πr^2^ and volume = 4π/3r^3^. *Recurrent platinum resistant intraperitoneal low passage patient derived model*: PS#3971 cells were isolated from a patient's ascites with recurrent SOC. The patient failed the next round of platinum therapy, and her cells were verified to be platinum resistant (Supplementary Figure S1). Cells were grown in standard adherent tissue culture conditions until a sufficient number of cells was obtained (<5 passages). Mice were randomized into equal weight groups. Three million PS#3971 cells were injected IP into nude mice, allowed to disseminate and grow for 7 days, and then daily gavaging and weighing began. Necramed (150mg/kg metformin, 50 mg/kg chloroquine, 25 mg/kg [5% human-mouse converted dose equivalent] nelfinavir, 2.25 mg/kg rapamycin, and 10 mg/kg dasatinib, in 50% PEG400/saline) or control (50% PEG400, 50% saline) gavaging was performed for 32 days. Mice were euthanized and visible tumors were harvested, photographed, weighed, and lysed for immunoblotting.

### Mouse safety models

Control (gavage, daily, 50% PEG400) or Necramed (chloroquine (30 mg/kg), nelfinavir (250 mg/kg), rapamycin (2.24 mg/kg), metformin (150 mg/kg), and dasatinib (4 mg/kg) in 50% PEG400, gavage, daily) treatment began at 10 weeks of age. For histology, nude mice were treated for 7 days and then euthanized 3 hours following the last treatment. Liver, heart, and kidneys were harvested, fixed in 10% buffered formalin for 24 hours and then 70% ethanol for at least 24 hours, blocked, sectioned with 3 μm width, and H&E stained by the core facility at the Moores Cancer Center. Histology images were taken with a 4X objective on a Nikon Eclipse TE2000E inverted microscope. An additional 3 pairs of C57BL/6 mice were treated with control or Necramed for 21 days, sacrificed, and blood collected immediately by cardiac extraction. Blood was centrifuged in a Micro Serum Separator (Professional Hospital Supply) and plasma saved in a microcentrifuge tube at 20°C until analysis by a VetScan instrument using a VS Complete Diagnostic Profile.

## SUPPLEMENTARY FIGURES AND TABLE


