# Supplementary material for: A strategy to combine pathway-targeted low toxicity drugs in ovarian cancer
Source: Oncotarget. 2015 Sep 22;6(31):31104–18. doi: 10.18632/oncotarget.5093 (PMC4741591; doi:10.18632/oncotarget.5093)
Supplement: Supplementary file 1 [file oncotarget-06-31104-s001.pdf]

SUPPLEMENTARY FIGURES AND TABLE

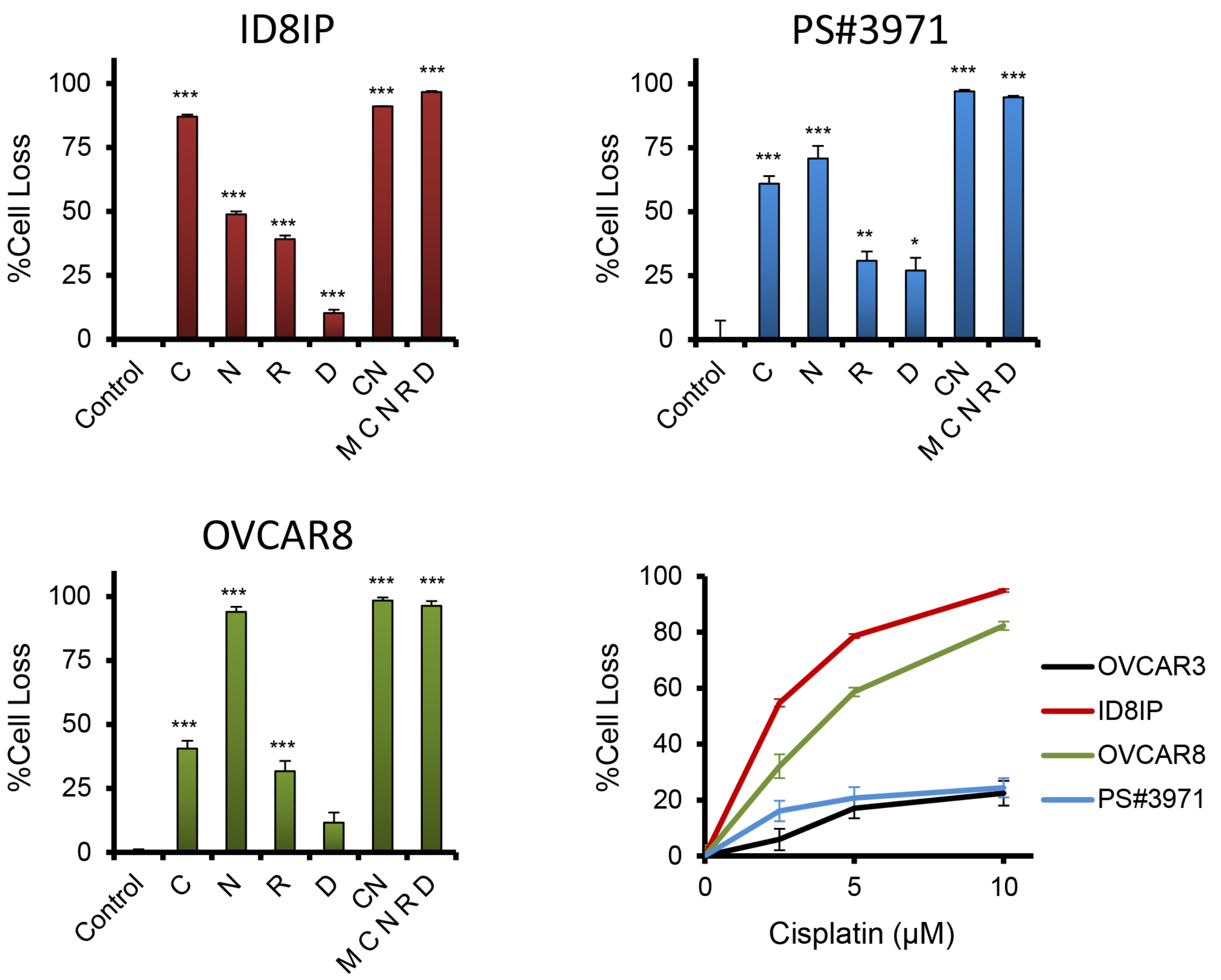

Supplementary Figure S1: Multiple SOC are sensitive to autophagic drugs. Crystal violet proliferation assay of indicated cell lines following 48 hours of treatment. Peak doses (Metformin, M 10 µM, Chloroquine, C 10 µM, Nelfinavir, N 10 µM, Rapamycin, R 10 nM, Dasatinib, D 50 nM) were used. *$p < 0.05$, **$p < 0.01$, ***$p < 0.001$ by *t*-test. Error bars are s.e.m.

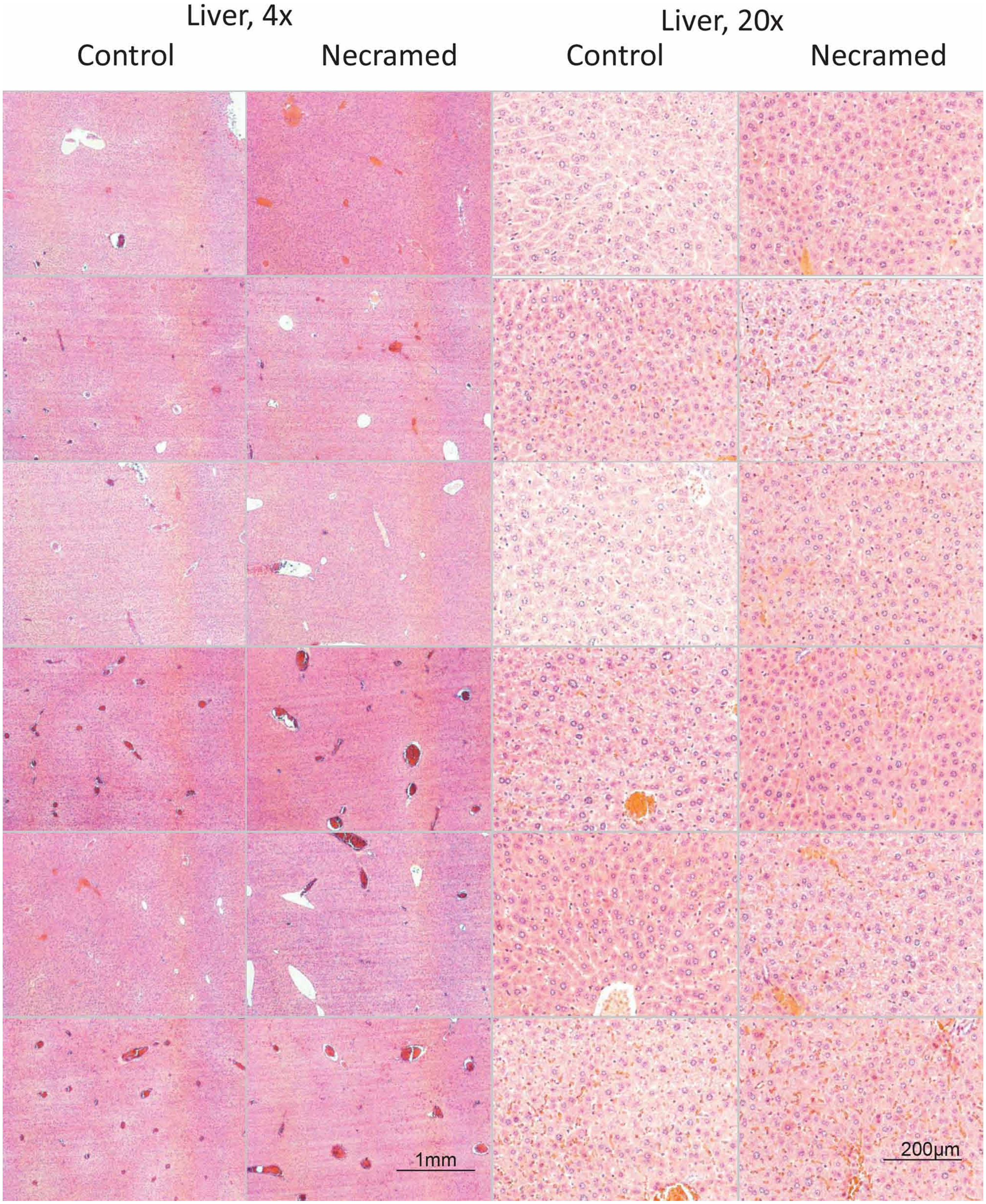
Liver, 4x
Control
Necramed
Liver, 20x
Control
Necramed
1mm
200µm

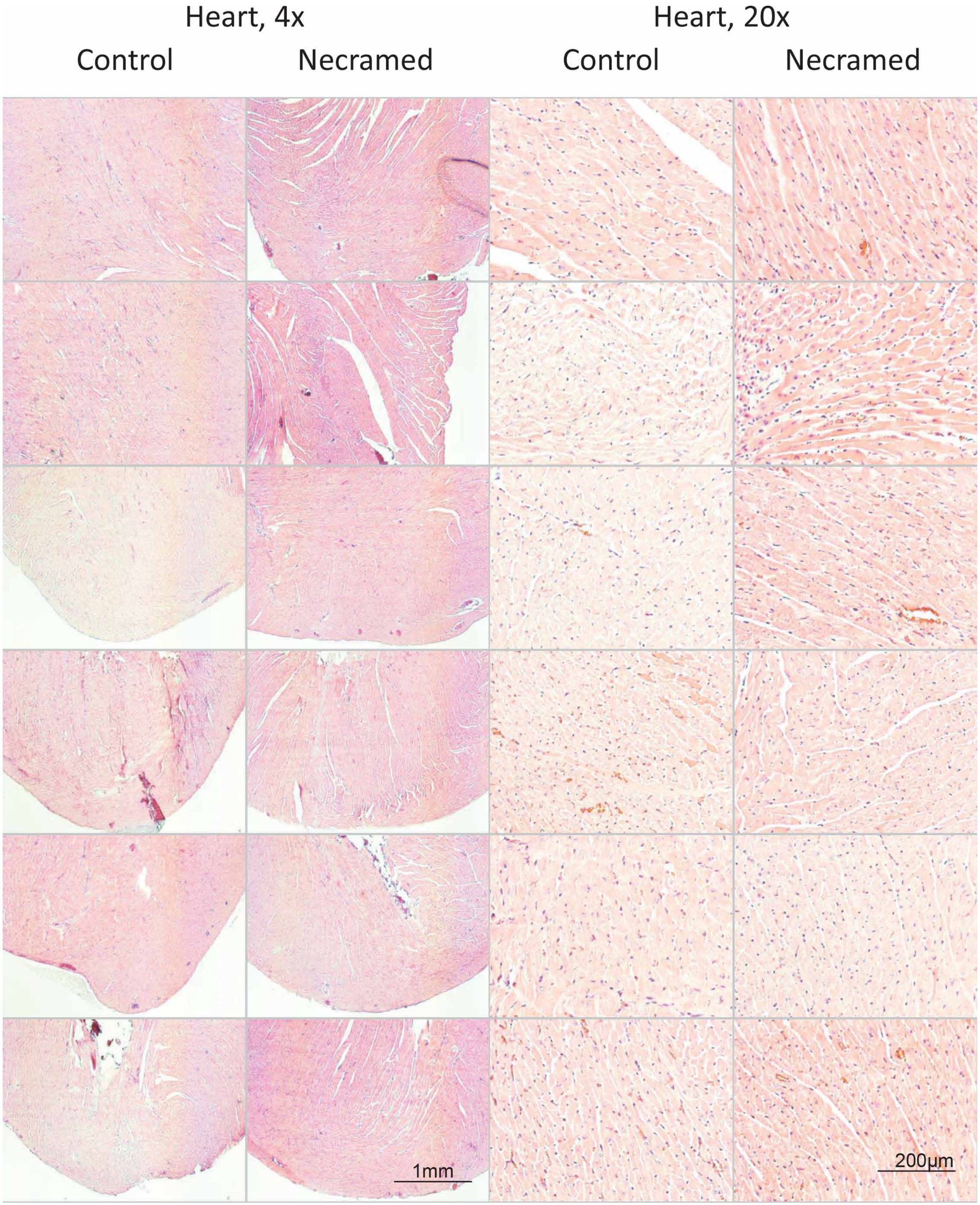
Heart, 4x
Control
Necramed
Heart, 20x
Control
Necramed
1mm
200µm

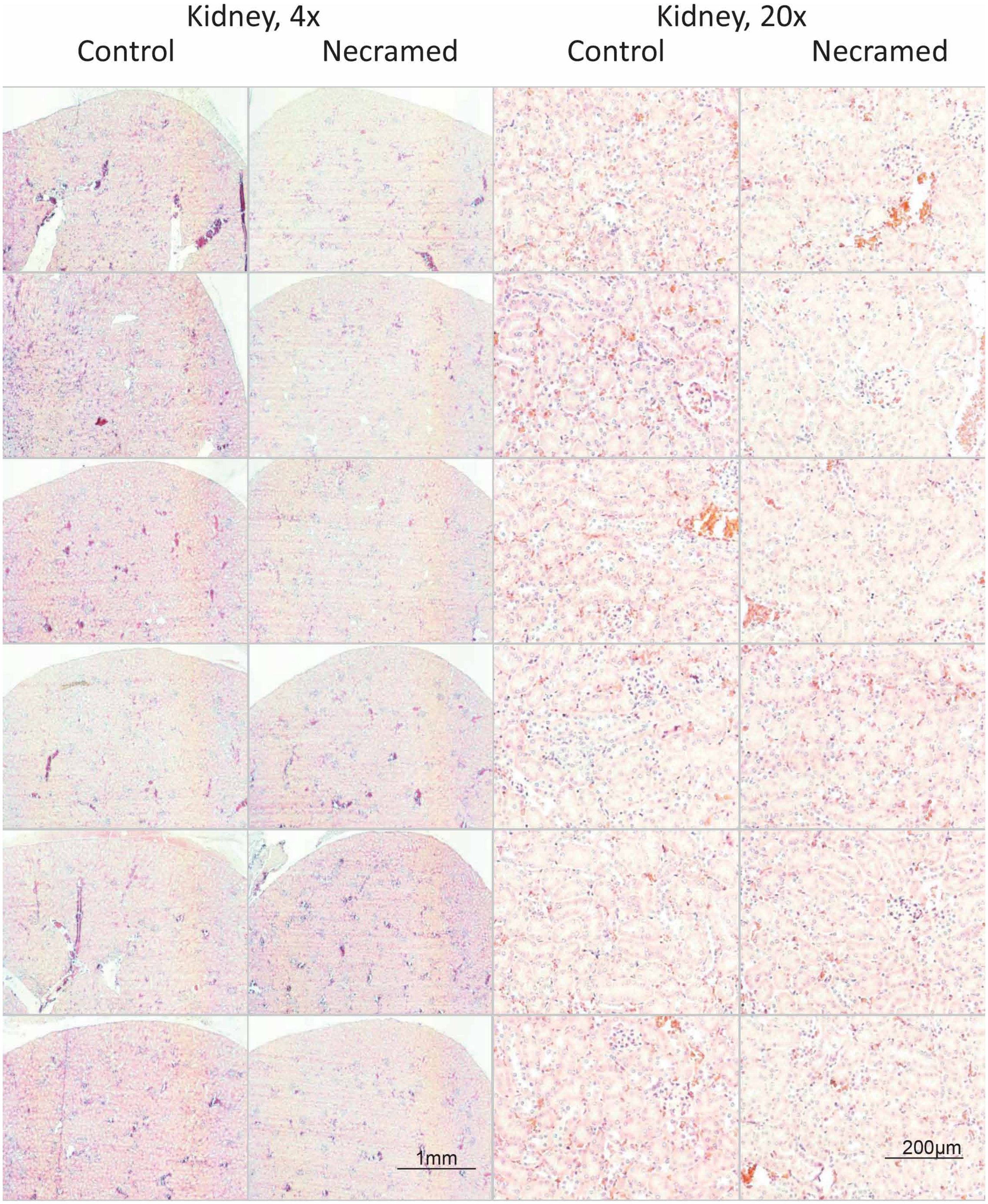

Supplementary Figure S2: Histology of heart, liver, and kidney from Necramed treated mice. Nude mice were treated for one week with Necramed, euthanized, and tissues harvested for H&E staining, as shown.

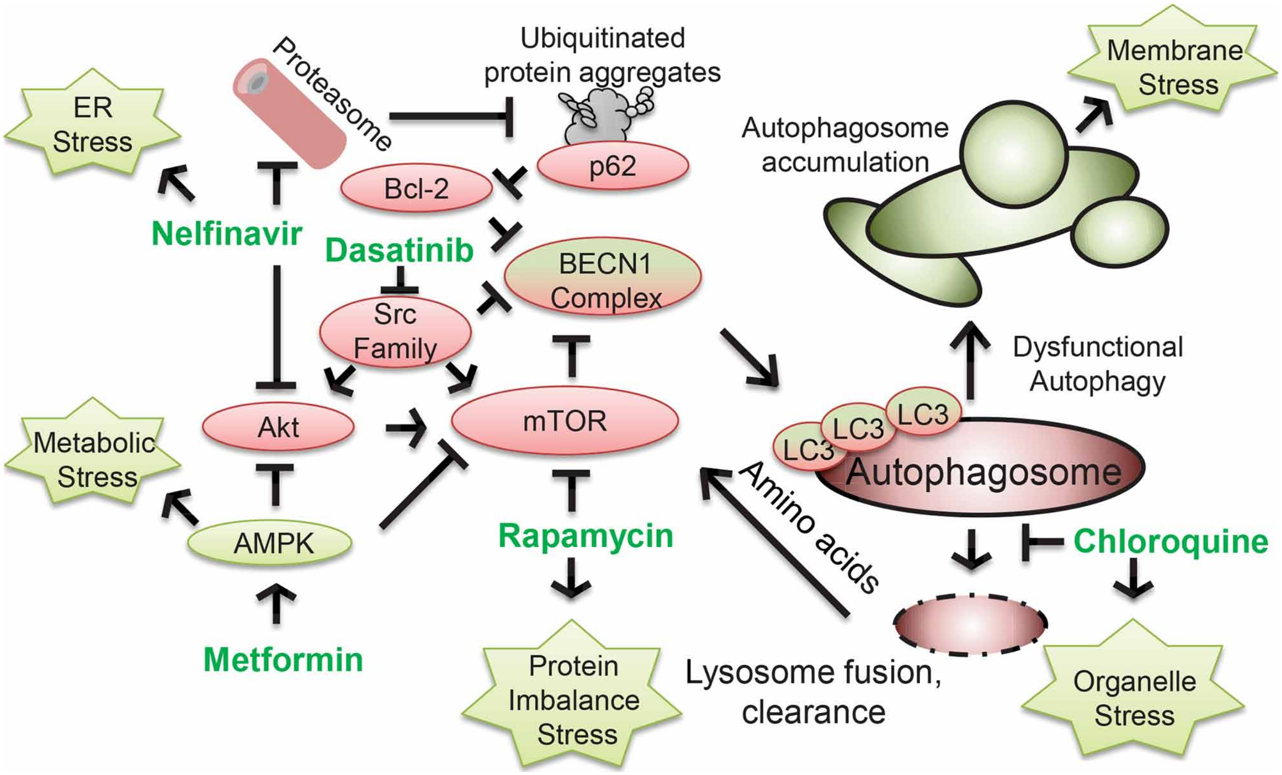

Supplementary Figure S3: Mechanisms of selected drugs in relation to autophagy.

Supplementary Table S1: Blood Chemistry Panels for Control- or Necramed-Treated Mice

Test		Unit	Range	Control			Necramed			*p* value
Albumin	ALB	g/dL	2.5–4.8	4.2	4.1	3.6	3.6	3.6	4.4	0.775
Alkaline Phosphatase	ALP	U/L	62–209	89	84	65	78	74	105	0.632
Alanine Transaminase	ALT	U/L	28–132	41	40	53	55	115	143	0.145
Amylase	AMY	U/L	1691–3615	1000	858	733	895	930	1051	0.364
Bilirubin, total	TBIL	mg/dL	0.1–0.9	0.4	0.4	0.3	0.3	0.5	0.4	0.649
Blood Urea Nitrogen	BUN	mg/dL	18–29	21	22	19	15	14	18	0.032
Calcium	CA	mg/dL	5.9–9.4	11.2	10.6	11.8	11.3	11.1	12.4	0.495
Phosphorus	PHOS	mg/dL	6.1–10.1	10.4	10.9	13.9	11.8	8.6	11	0.434
Creatinine	CRE	mg/dL	0.2–0.8	0.4	<0.2	0.3	0.4	<0.2	<0.2	0.608
Glucose	GLU	mg/dL	90–192	262	181	141	186	234	197	0.796
Sodium	NA+	mmol/L	126–182	155	162	160	161	159	160	0.684
Potassium	K+	mmol/L	4.7–6.4	>8.5	>8.5	>8.5	>8.5	8.5	>8.5	1.000
Total Protein	TP	g/dL	3.6–6.6	5.7	5.2	5.7	5.2	5.9	6.2	0.540
Globulin, calculated	GLOB	g/dL	N/A	1.5	1.1	2.2	1.6	2.3	1.8	0.484

Mice were treated daily for 21 days with Necramed or PEG/diluent (as described in *Methods*). Blood was collected by cardiac puncture and sent for evaluation of analytes. Final concetrations were expressed as grams or milligrams per deciliter (g/dL or mg/dL), units per liter, (U/L) or millimoles per liter (mmol/L).
